# AIM for Allostery: Using the Ising Model to Understand Information Processing and Transmission in Allosteric Biomolecular Systems

**DOI:** 10.3390/e17052895

**Published:** 2015-05-07

**Authors:** Michael V. LeVine, Harel Weinstein

**Affiliations:** 1 Department of Physiology and Biophysics, Weill Cornell Medical College, Cornell University, New York, NY 10065, USA; 2 HRH Prince Alwaleed Bin Talal Bin Abdulaziz Alsaud Institute of Computational Biomedicine, Weill Cornell Medical College, Cornell University, New York, NY 10065, USA

**Keywords:** allostery, biophysics, Ising model, statistical mechanics, signal transduction, information theory, G protein coupled receptors (GPCRs), dopamine D2 receptor, functional selectivity

## Abstract

In performing their biological functions, molecular machines must process and transmit information with high fidelity. Information transmission requires dynamic coupling between the conformations of discrete structural components within the protein positioned far from one another on the molecular scale. This type of biomolecular “action at a distance” is termed *allostery*. Although allostery is ubiquitous in biological regulation and signal transduction, its treatment in theoretical models has mostly eschewed quantitative descriptions involving the system's underlying structural components and their interactions. Here, we show how Ising models can be used to formulate an approach to allostery in a structural context of interactions between the constitutive components by building simple allosteric constructs we termed *Allosteric Ising Models* (AIMs). We introduce the use of AIMs in analytical and numerical calculations that relate thermodynamic descriptions of allostery to the structural context, and then show that many fundamental properties of allostery, such as the multiplicative property of parallel allosteric channels, are revealed from the analysis of such models. The power of exploring mechanistic structural models of allosteric function in more complex systems by using AIMs is demonstrated by building a model of allosteric signaling for an experimentally well-characterized asymmetric homodimer of the dopamine D2 receptor.

## 1. Introduction

Complex molecular assemblies and networks of interacting biomolecules mediate many cellular processes, such as cell growth, metabolism, and signaling. Molecular components of such assemblies and networks have been visualized and structurally elucidated at atomic-level resolution with experimental techniques including x-ray crystallography [1], nuclear magnetic resonance (NMR) [2], and cryo-electron microscopy (cryo-EM) [3]. The combination of structure elucidation with the application of biophysical methods reporting on the dynamic properties of the molecules (e.g., single molecule Forster resonance energy transfer (smFRET) [4], electron paramagnetic resonance (EPR) [5], Molecular Dynamics (MD) simulations [6], and elastic network models [7]) has produced detailed information regarding functional mechanisms. The application of these powerful methods of molecular biophysics has illuminated, especially in proteins, the large ensemble of conformations involved in the functional mechanisms of biomolecules, and hence the importance of conformational entropy. This conformational entropy is much higher than expected from crystal structures alone, and the relatively discrete structural elements comprising these systems (*i.e.*, loops, α-helices, β-strands, and a large number of tertiary structures in proteins) often exhibit coupled conformational dynamics. These coupled dynamics are especially crucial in receptor proteins, which are used to process and transmit information in their signaling function. For example, transmembrane receptor proteins, such as the G protein coupled receptors (GPCRs), bind extracellular ligands that trigger receptor “activation”, which is reflected by a change in conformation on the intracellular side of the protein where the transduction of the signal into the cell is accomplished [8]. This type of “action-at-a-distance” in the modulation of a specific function is referred to as allostery [9]. While allostery has been documented in many systems and has been suggested to be present in nearly all proteins [10], it is still unclear how most allosteric mechanisms work at the molecular level. A strong theoretical basis for allostery is needed, however, because such mechanisms are ubiquitous and essential for the transduction of signals and transmitting information both within proteins and throughout cellular systems. In addition, while there has been some success in engineering allosteric proteins from pre-existing components and scaffolds, a lack of detailed understanding has placed *de novo* design out of reach [11].

**Figure 1:**
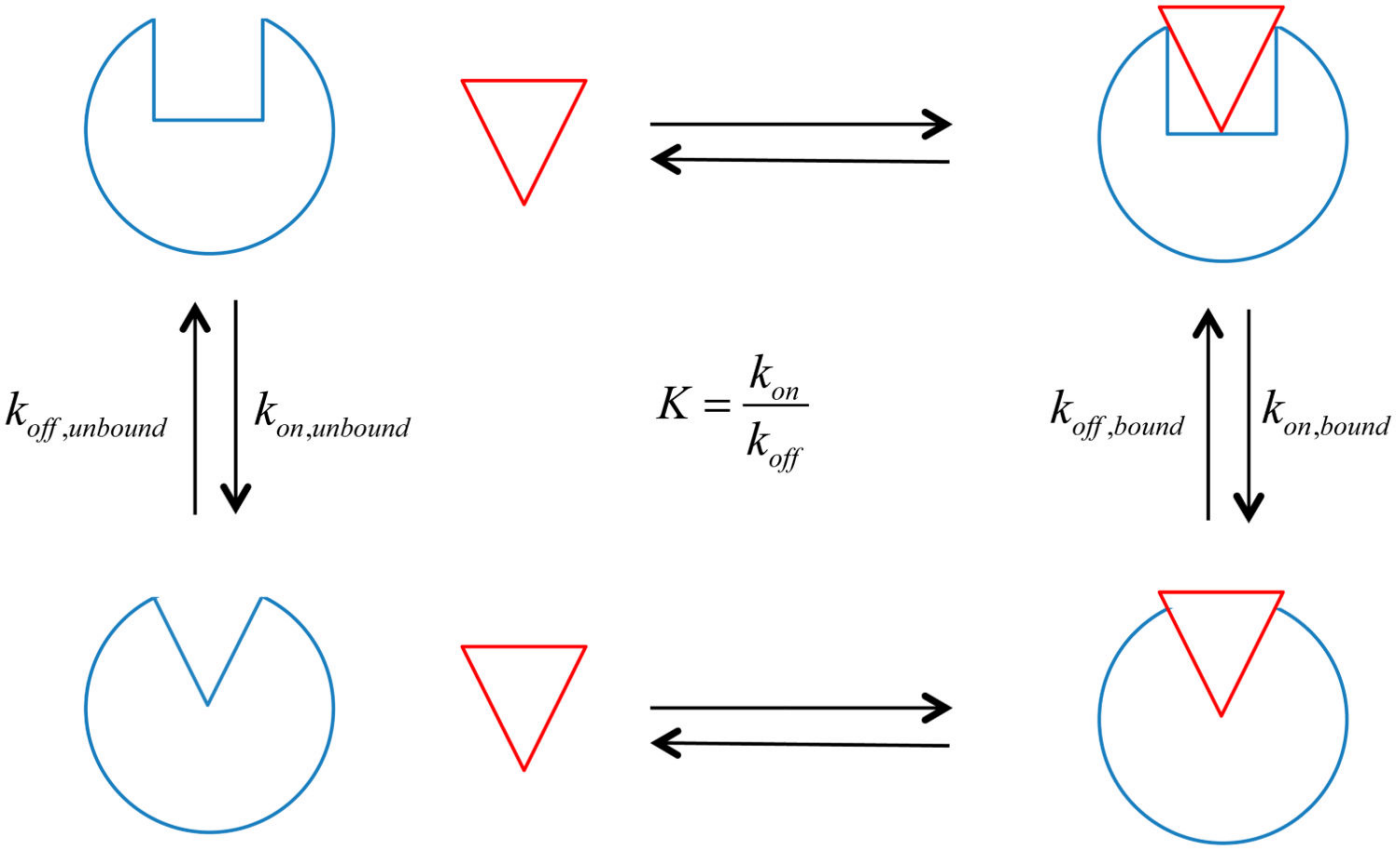
Thermodynamic cycle of a two-state ligand/receptor activation reaction. The receptor (blue circle) has an *on* and an *off* state (square and triangle indentations, respectively), both of which can bind a ligand (red triangle). The kinetic parameters are shown for the two equilibria of interest.

Considerations of theoretical models of allostery have generally followed a thermodynamic approach [9,12,13]. When biochemical measurements of the functional output of proteins can be made, the allosteric efficacy [14], which has also been called the allosteric coupling constant [15], can be used as a good measure of a ligand's allosteric influence on the protein's functional state. For the case of receptors, this downstream signal transduction can be measured experimentally. Assuming that the receptor has two states, *on* and *off*, an allosteric efficacy, α, can be defined as:

(1) $$\alpha =\frac{K_{\text{bound}}}{K_{\text{unbound}}}$$

where K_bound_ and K_unbound_ are the equilibrium constants for the activation reactions of the receptor when bound or unbound to the allosteric ligand, respectively. An equilibrium constant can be defined in terms of concentrations or rate constants:

(2) $$K=\frac{\left[R_{\text{on}}\right]}{\left[R_{\text{off}}\right]}=\frac{k_{\text{on}}}{k_{\text{off}}}$$

where [R_on_] and [R_off_] are the steady state concentrations of the receptor in the *on* and *off* state, respectively, and k_on_ and k_off_ are the corresponding rate constants for the transition to the *on* and *off* states (see Figure 1). The concentrations of the two receptor populations can be inferred from biochemical measurements of function, and the allosteric efficacy of the ligand of interest can be calculated from (1) and (2). When α > 1, the *on* state of the receptor is preferred in the presence of ligand and the ligand is considered an agonist (activator of function), and when α < 1, the *off* state of the receptor is preferred in the presence of ligand and the ligand is considered an inverse agonist (inhibitor of function). When α is 1, the ligand has no effect on the functional state of the receptor and the ligand is considered a neutral antagonist (inhibitor of activation by another ligand). This type of allostery, in which the equilibrium constant is modified by the ligand, is often described as “K-type”, as opposed to those that change enzyme catalysis in terms of k_cat_ or V_max_, which are described as “V-type” [15].

It is possible to conceptualize the allosteric efficacy of a ligand as a steady state signal-to-noise ratio, where the signal for the presence of a ligand in the binding site is encoded in the receptor on/off equilibrium constant that is sensed by the intracellular proteins that detect the signal by interacting with the receptor population. In the absence of ligand the equilibrium constant is non-zero (*i.e*., the probability of the receptor being active is non-zero), creating noise.

To obtain a formal definition of the allosteric efficacy in this context, it is possible to write the signal-to-noise ratio, SNR, as:

(3) $$\text{SNR}=\frac{P_{\text{signal}}}{P_{\text{noise}}}$$

where P_x_ is the power of x defined as:

(4) $$P_{x}=\frac{1}{T}\int_{0}^{T}x{\left(t\right)}^{2}\text{dt}$$

Therefore, the power of the equilibrium constant signal can be written as:

(5) $$P_{K}=\frac{1}{T}\int_{0}^{T}K{\left(t\right)}^{2}\text{dt}$$

and because at steady state the equilibrium constant is invariant with time by definition:

(6) $$P_{K}=K^{2}$$

then:

(7) $$\alpha =\sqrt{\frac{P_{K_{\text{bound}}}}{P_{K_{\text{unbound}}}}}$$

Accordingly, the allosteric efficacy of an agonist is a measure of the signal-to-noise ratio of signaling through the receptor with that agonist. If the ligand is an inverse agonist, the pertinent measure is the equilibrium constant for the inactivation reaction, so that the signal-to-noise ratio is simply α^-1^. When both the signal and noise are Gaussian, the Shannon-Hartley theorem [16,17] relates the signal-to-noise ratio to the information theoretical channel capacity C (which is the upper limit on the information rate or mutual information), by:

(8) C=Blog(1+SNR)

where B is the bandwidth of the channel. While Equation (8) is not directly applicable to the allosteric efficacy, as the signal and noise are not Gaussian, the treatment of allostery as an information transmission process has had much success recently [18–20], and we will confirm a strong relationship between the mutual information and allosteric efficacy later in the manuscript.

An energy-based expression of the allosteric efficacy can be written as the difference in free energies, G, of the four respective states:

(9) $$-\text{RT}\log(\alpha )=(G_{\text{on},\text{bound}}-G_{\text{on},\text{unbound}})+(G_{\text{off},\text{unbound}}-G_{\text{off},\text{bound}})$$

where R is the gas constant and T is the temperature. This model can be extended to systems with multiple ligand binding sites and/or allosterically regulated sites (for a detailed review, see [13]), but it clearly provides only a phenomenological explanation of allostery. According to this description, often considered “the thermodynamic” perspective, allostery occurs because of the differences in free energy of the respective states. However, this conclusion appears to be a definition, *i.e.*, that allostery is the phenomenon in which the stability of the *on* state relative to the *off* state is greater when the ligand is bound, and lesser when the ligand is unbound. From a “structural” perspective, one needs to consider the differences in free energy as emerging from some feature of the underlying network of interacting structural components, and it is this feature that makes the system allosteric.

To understand allostery at a level that explains the structural context for how allosteric biomolecular systems work requires a quantitative theoretical description that bridges the features of the structural components and their interactions, to the thermodynamic allosteric parameters. We address this problem in the next section.

## 2. Results and Discussion

### 2.1. The Thermodynamic Allosteric Efficacy as a Function of Local Interactions

We approach the problem of “how allostery works” by studying the statistical mechanics of interacting structural components. These structural components may be any subset of a biomolecular system that can be treated as a unit when described at some level of coarse-graining (*i.e*., a helix, a β strand, a helical bundle, a binding site, *etc*). The approach we will pursue is conceptually similar to the ensemble allosteric model (EAM) [12], but with the goal of introducing a structural context that can be analyzed analytically. Defining an n-component system X where for a single configuration each component can be in one of an arbitrary number of discrete states, we write the potential energy function of a given configuration of X, U(X), as:

(10) $$U(X)=\sum_{i=1}^{n}U^{\text{conf}}(X_{i})+\sum_{i=1}^{n}\sum_{j=1}^{n}\frac{U^{\mathrm{int}}(X_{i},X_{j})}{2}$$

The first term in (10) represents the conformational energy of each state of each component independent of other components, and the second term represents the pairwise interaction energy between components; all interaction terms when i = j are 0. We can write the probability of any conformation of the system according to the Boltzman distribution as:

(11) $$p(X)=\frac{e^{-\beta U(X)}}{Z}$$

β is 1/k_B_T, where k_B_ is the Boltzmann constant and T is the temperature in Kelvin. The numerator is known as the Boltzmann factor, and Z is the partition function, which sums over the Boltzmann factors of all states and normalizes the probability:

(12) $$Z=\sum e^{-\beta U(X)}$$

We can then define the specific case of ligand binding to a two-state receptor. This system can be defined as a two-component system in which each component is two-state: one component representing the receptor, R, with states *on* and *off*, and the second component representing the ligand, L, with states *bound* and *unbound*. It should be noted that for the ligand, the conformational energy term represents the component of the binding energy that is independent of the state of the receptor. Using the explicit definition of the concentration:

(13) $$[X]=\frac{N_{x}}{V}$$

where N_x_ is the number of molecules of X and V is the volume, we can rewrite (2) with the explicit definition of protein concentration:

(14) $$K=\frac{\frac{{\text{Nf}}_{\text{on}}}{V}}{\frac{{\text{Nf}}_{\text{off}}}{V}}=\frac{f_{\text{on}}}{f_{\text{off}}}$$

where N is the total number of receptors and f*_on_* and f*_off_* are the fraction of receptors in the *on* and *off* states, respectively. Given that the system is ergodic, the frequency of a given state at steady state will converge to the ensemble probabilities. Rewriting (1) by substituting thermodynamic equilibrium constants with ratios of probabilities, we can define the allosteric efficacy as:

(15) $$\alpha \frac{p(L=\text{unbound},R=\text{on})}{p(L=\text{unbound},R=\text{off})}=\frac{p(L=\text{bound},R=\text{on})}{p(L=\text{bound},R=\text{off})}$$

Using (10) and (11), we can write (15) as:

(16) $$\alpha \frac{e^{-\beta [U^{\text{conf}}(L=\text{unbound})+U^{\text{conf}}(R=\text{on})+U^{\mathrm{int}}(L=\text{unbound},R=\text{on})]}}{e^{-\beta [U^{\text{conf}}(L=\text{unbound})+U^{\text{conf}}(R=\text{off})+U^{\mathrm{int}}(L=\text{unbound},R=\text{off})]}}=\frac{e^{-\beta [U^{\text{conf}}(L=\text{bound})+U^{\text{conf}}(R=\text{on})+U^{\mathrm{int}}(L=\text{bound},R=\text{on})]}}{e^{-\beta [U^{\text{conf}}(L=\text{bound})+U^{\text{conf}}(R=\text{off})+U^{\mathrm{int}}(L=\text{bound},R=\text{off})]}}$$

Equation (16) reduces to:

(17) $$\alpha =e^{-\beta [(U^{\mathrm{int}}(L=\text{bound},R=\text{on})-U^{\mathrm{int}}(L=\text{bound},R=\text{off}))+(U^{\mathrm{int}}(L=\text{unbound},R=\text{off})-U^{\mathrm{int}}(L=\text{unbound},R=\text{on}))]}$$

We then find the analogous expression of (9):

(18) $$-\frac{1}{\beta }\log(\alpha )=(U^{\mathrm{int}}(L=\text{bound},R=\text{on})-U^{\mathrm{int}}(L=\text{bound},R=\text{off}))+(U^{\mathrm{int}}(L=\text{unbound},R=\text{off})-U^{\mathrm{int}}(L=\text{unbound},R=\text{on}))$$

As (18) indicates, the allosteric efficacy is a function the interaction energy between the states, and we have succeeded in expressing the thermodynamic allosteric efficacy as a function of local interactions in our simple two-component ligand/receptor system. However, this result is significantly more useful for considering multi-component systems if additional energetic symmetries are imposed by using an Ising model potential energy function. While these symmetries are not strictly realized in a biomolecular system, we will show that their application leads to concise analytic expressions that are qualitatively and quantitatively accurate as well for systems in which these symmetries are not present.

### 2.2. The Allosteric Ising Model (AIM) for Multicomponent Systems

The Ising model is a statistical mechanical model originally developed to describe phase behavior in ferromagnetic materials [21]. The Ising model, as well as Ising-like models, have since been applied to other complex systems with collective behavior [22,23], including cooperativity during folding [24–26] and in oligomeric assemblies [27,28]. In the Ising model, each particle has two states, corresponding to a spin state of up or down:

(19) $$s_{x}=\{\begin{array}{cc} -1 & X=↓\\ 1 & X=↑ \end{array}$$

The potential energy function of an n-component Ising model is:

(20) $$U(X)=-\sum_{i=1}^{n}h_{i}s_{i}-\sum_{i=1}^{n}\sum_{j=1}^{n}\frac{j_{ij}}{2}s_{i}s_{j}$$

In the Ising model, hi is the potential energy of particle i due to the magnetic field, and j_ij_ is the spin coupling between particles i and j, where j_ii_ is taken to be 0. If the field term is taken to be site-specific, one can see that the field term can be considered to correspond to the conformational energy, and the spin coupling term to the pairwise interaction energy. We can rewrite the potential function as:

(21) $$U(X)=\sum_{i=1}^{n}u_{i}^{\text{conf}}s_{i}+\sum_{i=1}^{n}\sum_{j=1}^{n}\frac{u_{i,j}^{\mathrm{int}}}{2}s_{i}s_{j}$$

where $u_{i}^{\text{conf}}$ is the conformational energy of component i, and $u_{i,j}^{\mathrm{int}}$ is the interaction energy of components i and j. By using (21) for the potential energy function, we impose the following symmetries on the two-state components (with binary states represented by up and down arrows):

(22) $$\begin{array}{c} U^{\text{conf}}(X=↑)=-U^{\text{conf}}(X=↓)\\ U^{\mathrm{int}}(X_{i}=↑,X_{j}=↑)=U^{\mathrm{int}}(X_{i}=↓,X_{j}=↓)=-U^{\mathrm{int}}(X_{i}=↑,X_{j}=↓)=-U^{\mathrm{int}}(X_{i}=↓,X_{j}=↑) \end{array}$$

For Ising models composed of several components and various interaction topologies, these symmetries allow for concise analytical expression for the allosteric efficacy and binding affinity. We will refer to these models as Allosteric Ising Models (AIMs).

**Figure 2:**
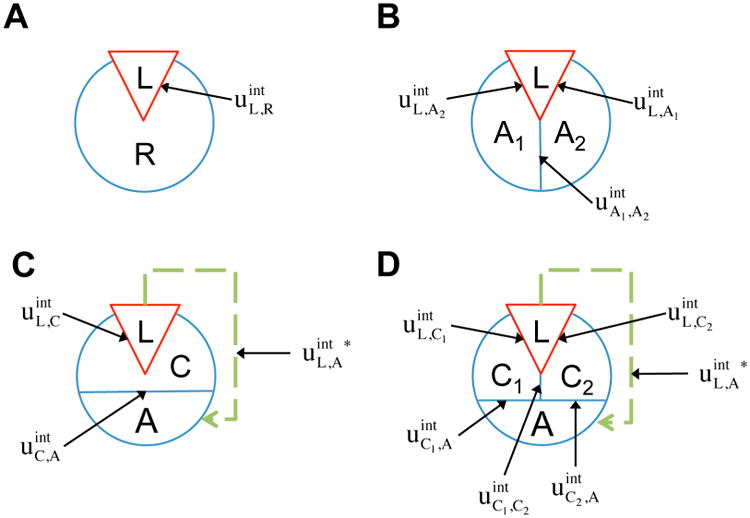
Schematic representations of Allosteric Ising models (AIMs). In the four AIMs analyzed here the ligand, L, is represented as a red triangle, and the protein is the blue circle subdivided into various constituent structural components. Lines separating ligand from protein or protein structural components from each other are labeled with the appropriate interaction energy term (as used in the text). Allosteric effective interactions are represented with green dotted lines. (**A**): The simple two-component ligand/receptor system. (**B**): A three-component ligand/receptor system with two allosteric sites, A_1_ and A_2_. (**C**): A three-component ligand/receptor system with one channel, C, coupling the ligand and the allosteric site A. (**D**): A four-component ligand/receptor system with two channels, C1 and C2, coupling the ligand and the allosteric site A.

Considering the analogy to the ligand(L)-receptor(R) systems and treating the *on*/*off* and *bound*/*unbound* states as *up*/*down* spins (see Figure 2A), the potential energy function according to (21) can be written as:

(23) $$U(s_{L},s_{R})=u_{L}^{\text{conf}}s_{L}+u_{R}^{\text{conf}}s_{R}+u_{L,R}^{\mathrm{int}}s_{L}s_{R}$$

As the interaction energy between the receptor and the ligand must be zero when the ligand is in the unbound state, we write an alternative non-Ising potential energy function where the interaction energy is 0 when the ligand is unbound:

(24) $$U(s_{L},s_{R})=u_{L}^{\text{conf}}s_{L}+u_{R}^{\text{conf}}s_{R}+u_{L,R}^{\mathrm{int}}\frac{s_{L}+1}{2}s_{R}$$

This equation can be re-written as an Ising model potential energy function:

(25) $$U(s_{L},s_{R})=u_{L}^{\text{conf}}s_{L}+(u_{R}^{\text{conf}}+\frac{u_{L,R}^{\mathrm{int}}}{2})s_{R}+\frac{u_{L,R}^{\mathrm{int}}}{2}s_{L}s_{R}$$

Thus we will proceed with (23) despite the seemingly non-physical interaction, and later confirm that the relationships derived using this model accurately represent those of non-Ising systems. The allosteric efficacy using this potential energy function is:

(26) $$\alpha \frac{p(L=↓,R=↑)}{p(L=↓,R=↓)}=\frac{p(L=↑,R=↑)}{p(L=↑,R=↓)}$$

and we can simplify (17) to:

(27) $$\alpha =e^{-4\beta u_{L,R}^{\mathrm{int}}}$$

Equation (27) indicates that in the Allosteric Ising Model for the ligand/receptor system (“ligand/receptor AIM”), the allosteric efficacy is simply a function of the ligand-receptor interaction energy term. Positive allostery (agonism) is attributed to negative interaction energy; negative allostery (inverse agonism) is attributed to positive interaction energy. Note that as the interaction energy between the ligand and receptor is related to the allosteric efficacy by a log transformation, we will use here the allosteric efficacy and interaction energy interchangeably, and specifically use interaction energy for visual representations, where the log scale is required.

The two-component model assumes that the protein is entirely rigid, with two global states. However, it is possible for the ligand to allosterically modulate multiple distinct allosteric sites (see Figure 2B). It is well known, for example, that GPCRs can signal through multiple downstream signaling pathways through coupling to various G protein subtypes and β-arrestin [29,30], and that different ligands can differentially activate these pathways [31,32]. Therefore it may be necessary to distinguish among multiple allosteric sites in the representation of receptor allostery. If we introduce two non-interacting allosteric sites, A_1_ and A_2_, we can write the potential energy function as:

(28) $$U(L,A_{1},A_{2})=u_{L}^{\text{conf}}+u_{A_{1}}^{\text{conf}}+u_{A_{2}}^{\text{conf}}+u_{L,A_{1}}^{\mathrm{int}}+u_{L,A_{2}}^{\mathrm{int}}$$

Then the allosteric efficacy at a site is:

(29) $$\alpha \frac{p(L=↓,A_{1}=↑)}{p(L=↓,A_{1}=↓)}=\frac{p(L=↑,A_{1}=↑)}{p(L=↑,A_{1}=↓)}$$

The probability of each state is the sum of the probability of two underlying states:

(30) $$\alpha_{L,A_{1}}\frac{p(L=↓,A_{1}=↑,A_{2}=↑)+p(L=↓,A_{1}=↑,A_{2}=↓)}{p(L=↓,A_{1}=↓,A_{2}=↑)+p(L=↓,A_{1}=↓,A_{2}=↓)}=\frac{p(L=↑,A_{1}=↑,A_{2}=↑)+p(L=↑,A_{1}=↑,A_{2}=↓)}{p(L=↑,A_{1}=↓,A_{2}=↑)+p(L=↑,A_{1}=↓,A_{2}=↓)}$$

which is equal to:

(31) $$\alpha_{L,A_{1}}\frac{e^{-\beta [-u_{L}^{\text{conf}}+u_{A_{1}}^{\text{conf}}+u_{A_{2}}^{\text{conf}}-u_{L,A_{1}}^{\mathrm{int}}-u_{L,A_{2}}^{\mathrm{int}}]}+e^{-\beta [-u_{L}^{\text{conf}}+u_{A_{1}}^{\text{conf}}-u_{A_{2}}^{\text{conf}}-u_{L,A_{1}}^{\mathrm{int}}+u_{L,A_{2}}^{\mathrm{int}}]}}{e^{-\beta [-u_{L}^{\text{conf}}-u_{A_{1}}^{\text{conf}}+u_{A_{2}}^{\text{conf}}+u_{L,A_{1}}^{\mathrm{int}}-u_{L,A_{2}}^{\mathrm{int}}]}+e^{-\beta [-u_{L}^{\text{conf}}-u_{A_{1}}^{\text{conf}}-u_{A_{2}}^{\text{conf}}+u_{L,A_{1}}^{\mathrm{int}}+u_{L,A_{2}}^{\mathrm{int}}]}}=\frac{e^{-\beta [u_{L}^{\text{conf}}+u_{A_{1}}^{\text{conf}}+u_{A_{2}}^{\text{conf}}+u_{L,A_{1}}^{\mathrm{int}}+u_{L,A_{2}}^{\mathrm{int}}]}+e^{-\beta [u_{L}^{\text{conf}}+u_{A_{1}}^{\text{conf}}-u_{A_{2}}^{\text{conf}}+u_{L,A_{1}}^{\mathrm{int}}-u_{L,A_{2}}^{\mathrm{int}}]}}{e^{-\beta [u_{L}^{\text{conf}}-u_{A_{1}}^{\text{conf}}+u_{A_{2}}^{\text{conf}}+u_{L,A_{1}}^{\mathrm{int}}-u_{L,A_{2}}^{\mathrm{int}}]}+e^{-\beta [u_{L}^{\text{conf}}-u_{A_{1}}^{\text{conf}}-u_{A_{2}}^{\text{conf}}-u_{L,A_{1}}^{\mathrm{int}}-u_{L,A_{2}}^{\mathrm{int}}]}}$$

This reduces to:

(32) $$\alpha_{L,A_{1}}=e^{-4\beta u_{L,A_{1}}^{\mathrm{int}}}$$

which indicates that the allosteric efficacy of a ligand at an allosteric site is independent of other allosteric sites it modulates as well (provided the allosteric sites are not coupled through another interaction). In terms of receptor signaling, this analysis predicts that there could exist ligands with absolute bias for only one signaling pathway. This would require the downstream effectors (*e.g.,* the G proteins or β-arrestin for GPCRs) to interact with unique and independent allosteric sites.

### 2.3. Representation of allosteric propagation through specific regions within the protein

In addition to the existence of multiple allosteric sites, allosteric conformational coupling can be propagated through specific regions within the protein, often called “paths” or “channels”. Using the AIM approach described here, we can expand the treatment of allostery to proteins with multiple structural components, where some components are allosterically regulated, and some others mediate the allosteric regulation. We begin with a three-component model, composed of the ligand L, a channel C, and an allosteric site A (see AIM represented in Figure 2C). The potential energy function is:

(33) $$U(L,C,A)=u_{L}^{\text{conf}}s_{L}+u_{C}^{\text{conf}}s_{C}+u_{A}^{\text{conf}}s_{A}+u_{L,C}^{\mathrm{int}}s_{L}s_{C}+u_{C,A}^{\mathrm{int}}s_{C}s_{A}+u_{L,A}^{\mathrm{int}}s_{L}s_{A}$$

The allosteric efficacy is then:

(34) $$\alpha_{L,A_{1}}\frac{e^{-\beta [-u_{L}^{\text{conf}}+u_{C}^{\text{conf}}+u_{A}^{\text{conf}}-u_{L,C}^{\mathrm{int}}-u_{L,A}^{\mathrm{int}}+u_{C,A}^{\mathrm{int}}]}+e^{-\beta [-u_{L}^{\text{conf}}+u_{C}^{\text{conf}}-u_{A}^{\text{conf}}-u_{L,C}^{\mathrm{int}}+u_{L,A}^{\mathrm{int}}-u_{C,A}^{\mathrm{int}}]}}{e^{-\beta [-u_{L}^{\text{conf}}-u_{C}^{\text{conf}}+u_{A}^{\text{conf}}+u_{L,C}^{\mathrm{int}}-u_{L,A}^{\mathrm{int}}-u_{C,A}^{\mathrm{int}}]}+e^{-\beta [-u_{L}^{\text{conf}}-u_{C}^{\text{conf}}-u_{A}^{\text{conf}}+u_{L,C}^{\mathrm{int}}+u_{L,A}^{\mathrm{int}}+u_{C,A}^{\mathrm{int}}]}}=\frac{e^{-\beta [u_{L}^{\text{conf}}+u_{C}^{\text{conf}}+u_{A}^{\text{conf}}+u_{L,C}^{\mathrm{int}}+u_{L,A}^{\mathrm{int}}+u_{C,A}^{\mathrm{int}}]}+e^{-\beta [u_{L}^{\text{conf}}+u_{C}^{\text{conf}}-u_{A}^{\text{conf}}+u_{L,C}^{\mathrm{int}}-u_{L,A}^{\mathrm{int}}-u_{C,A}^{\mathrm{int}}]}}{e^{-\beta [u_{L}^{\text{conf}}-u_{C}^{\text{conf}}+u_{A}^{\text{conf}}+u_{L,C}^{\mathrm{int}}-u_{L,A}^{\mathrm{int}}-u_{C,A}^{\mathrm{int}}]}+e^{-\beta [u_{L}^{\text{conf}}-u_{C}^{\text{conf}}-u_{A}^{\text{conf}}-u_{L,C}^{\mathrm{int}}-u_{L,A}^{\mathrm{int}}+u_{C,A}^{\mathrm{int}}]}}$$

Equation (34) simplifies to:

(35) $$\alpha_{L,A}=e^{-4\beta u_{L,A}^{\mathrm{int}}}\frac{\cosh(2\beta (u_{L,C}^{\mathrm{int}}+u_{C,A}^{\mathrm{int}}))+\cosh(2\beta u_{C}^{\text{conf}})}{\cosh(2\beta (u_{L,C}^{\mathrm{int}}-u_{C,A}^{\mathrm{int}}))+\cosh(2\beta u_{C}^{\text{conf}})}$$

where cosh is the hyperbolic cosine function:

(36) $$\cosh(x)=\frac{e^{x}+e^{-x}}{2}$$

It should be noted that the exponential term in (35) is the *conditional allosteric efficacy*. The conditional allosteric efficacy can be written as the sum of weighted allosteric efficacies, with each allosteric efficacy conditioned on a different state of the channel and then weighted by the corresponding probability of that state:

(37) $$\alpha_{L,A|C}=p(C=↑)\alpha_{L,A|C=↑}+p(C=↓)\alpha_{L,A|C=↓}$$

where for a given state, s, of C:

(38) $$\alpha_{L,A|C=s}=\frac{p(L=↑,A=↑,C=s)p(L=↓,A=↓,C=s)}{p(L=↑,A=↓,C=s)p(L=↓,A=↑,C=s)}$$

Equation (38) simplifies to:

(39) $$\alpha_{L,A|C}=e^{-4\beta u_{L,A}^{\mathrm{int}}}$$

Comparing (39) with the allosteric efficacy of the two-component ligand/receptor system expressed in (27), it is clear that the conditional allosteric efficacies in the three-component system are simply the allosteric efficacies of the corresponding two-component systems.

We can then differentiate the allosteric efficacy contributed by the direct interaction of two components, *i.e*., the conditional allosteric efficacy, from the indirect contributions, and write:

(40) $$\alpha_{L,A}=\alpha_{L,A|C}\alpha_{L,A}^{\text{indirect}}$$

where the allosteric efficacy contributed by the indirect interaction is:

(41) $$\alpha_{L,A}^{\text{indirect},C}=\frac{\cosh(2\beta (u_{L,C}^{\mathrm{int}}+u_{C,A}^{\mathrm{int}}))+\cosh(2\beta u_{C}^{\text{conf}})}{\cosh(2\beta (u_{L,C}^{\mathrm{int}}-u_{C,A}^{\mathrm{int}}))+\cosh(2\beta u_{C}^{\text{conf}})}$$

Importantly, (41) provides a description of the allosteric efficacy as a function of the channel through which it is propagated. There are immediate inferences that can be drawn from this representation. First, the channel must have little preference for either one of its conformations, so that signaling through it can have a high intrinsic signal-to-noise ratio. Based on this inference, mutations that further stabilize the intrinsically preferred conformation of a channel will decrease the allosteric efficacy of a ligand, whereas mutations that destabilize that conformation will increase the allosteric efficacy. The existence of these two classes of mutations has immediate implications for the ability to test experimentally the role of specific domains in allosteric signaling. Second, because allosteric transmission through the channel depends on a balance between the channel's conformational energy and the interaction energy between the channel and ligand, and the channel and allosteric site, it follows that a low intrinsic signal-to-noise ratio can be overcome by an increased coupling of the ligand to the channel. Lastly, if the sign of the coupling of the ligand to the channel is opposite that of the channel to the allosteric site, the allosteric signal can be reversed. Consequently, a binding site on a protein that has been evolved for positive allostery by endogenous ligands, can be targeted as a site for negative allosteric modulation, and *vice versa*. It is well known that endogenous agonist-binding sites can be targeted by inverse-agonists, so this result is anchored in experimental evidence.

### 2.4. The Channel as a Chain of Interacting Structural Components

Comparison of (35) with (39) indicates that the allosteric efficacy can be written in terms of the conditional allosteric efficacies due to direct interactions:

(42) $$\alpha_{L,A}=\alpha_{L,A|C}\frac{\cosh(\frac{1}{2}\log(\alpha_{L,C|A}\alpha_{C,A|L}))+\cosh(2\beta u_{C}^{\text{conf}})}{\cosh(\frac{1}{2}\log(\frac{\alpha_{L,C|A}}{\alpha_{C,A|L}}))+\cosh(2\beta u_{C}^{\text{conf}})}$$

In effect, the conditional allosteric efficacy is the signal-to-noise ratio for a single step in the signal propagation process, and the effective signal-to-noise ratio for the entire signal propagation system can be described by a non-linear function of all the constituent propagation steps.

Equation (42) can also be written as the effective interaction energy, $u_{L,A}^{\mathrm{int}*}$ :

(43) $$u_{L,A}^{\mathrm{int}*}=u_{L,A}^{\mathrm{int}}-\frac{1}{4\beta }\log(\frac{\cosh(2\beta (u_{L,C}^{\mathrm{int}}+u_{C,A}^{\mathrm{int}}))+\cosh(2\beta u_{C}^{\text{conf}})}{\cosh(2\beta (u_{L,C}^{\mathrm{int}}-u_{C,A}^{\mathrm{int}}))+\cosh(2\beta u_{C}^{\text{conf}})})$$

and thus as the sum of the direct and indirect interactions:

(44) $$u_{L,A}^{\mathrm{int}*}=u_{L,A}^{\mathrm{int}}+u_{L,A}^{\text{indirect},C}$$

It should be noted that the designation of channel versus allosteric site is purely an operational definition in which the site that performs the function of interest is referred to as the allosteric site. If both sites are functional, such as the example of two independent allosteric sites described above, and if they interact, we can rewrite (42) as:

(45) $$\alpha_{L,A_{1}}=\alpha_{L,A_{1}|A_{2}}\frac{\cosh(\frac{1}{2}\log(\alpha_{L,A_{2}|A_{1}}\alpha_{A_{1},A_{2}|L}))+\cosh(2\beta u_{A_{2}}^{\text{conf}})}{\cosh(\frac{1}{2}\log(\frac{\alpha_{L,A_{2}|A_{1}}}{\alpha_{A_{1},A_{2}|L}}))+\cosh(2\beta u_{A_{2}}^{\text{conf}})}$$

**Figure 3:**
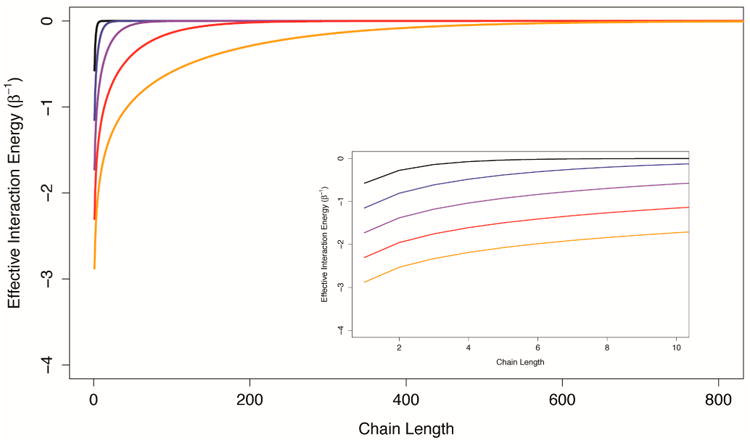
The effective interaction energy through serial channels. Effective interaction energies of the first and last components of one-dimensional Ising chains are plotted as a function of chain length for conditional allosteric efficacy values of 10 (black), 100 (blue), 1000 (purple) 10,000 (red) and 100,000 (orange). The inset shows detail for short chain lengths. The effective interaction energy is seen to decay exponentially with channel length.

The description of the allosteric efficacy as a function of the channel through which it is propagated, in (41), indicates that if the channel is a one-dimensional chain of interacting structural components, the allosteric efficacy is quickly diminished (it has been shown that the spin correlation function decays exponentially with distance in one-dimensional Ising models [21]). In Figure 3, the effective interaction energy between the first and last components of one-dimensional Ising chains with uniform conditional allosteric efficacies of 10, 100, 1,000, 10,000, and 100,000 are shown as a function of chain length. For weakly interacting systems, channels formed by structural components interacting in series do not appear to be good mediators of allosteric efficacy. The prevalence of multi-segment transmembrane signaling complexes may indicate an evolutionary mechanism to overcome the limitations of serial channels.

### 2.5. Comparison of Allosteric Propagation in Ising and Non-Ising Systems

As described in Section 2.2, the above analysis is made possible through the energetic symmetries imposed by the Ising model. However, it is unlikely these energetic symmetries exist in real allosteric proteins. Thus, it is important to consider how well the relationships derived from AIMs describe non-Ising two-state models, which are expected to be better representations of the types of interaction networks present in the biomolecular systems of interest.

To consider this problem, we sampled 100,000 non-Ising two-state allosteric systems with interaction energies and configurational energies sampled from normal distributions of mean 0 and standard deviation of β^−1^, 3/β, or 5/β. The exact allosteric efficacies, calculated from the exact probabilities of each state, were then compared to the allosteric efficacies estimated from (42) using the direct allosteric efficacy terms. We should note that while direct allosteric efficacies can be calculated for non-Ising model, the calculation of the configuration energy term followed:

(46) $$2u_{C}^{\text{conf}}\approx U^{\text{conf}}(C=↑)-U^{\text{conf}}(C=↓)$$

**Figure 4:**
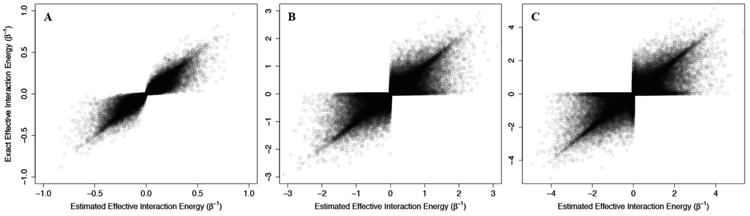
Using the Ising model to estimate effective interaction energies in non-Ising three-component/two-state systems. The exact effective interaction energies of 100,000 three-component/two-state non-Ising systems are plotted against the effective interaction energy estimated using the equations derived for the three-component Ising model (see (42)). The systems are generated using energy terms sampled from a normal distribution of mean 0 and standard deviation of 1/β (**A**), 3/β (**B**), and 5/β (**C**) and the points are plotted with 10% opacity.

As above, we addressed problems that may arise from the non-physical interaction energy between unbound ligand and the protein by setting to 0 all interaction energies with the unbound ligand. Results of these calculations are shown in Figure 4, where the corresponding effective interaction energies have been used for clarity. Our calculations indicate that (42) is a good estimate of the true allosteric efficacy in non-Ising systems in which the allosteric efficacy is high (see Figure 4A). As the standard deviation on the energy term distribution increases, and more systems have significant deviation from Ising-like behavior, two distinct groups of false positives (exact effective interaction energy is 0 but estimated interaction energy is non-zero) and true negatives (exact effective interaction energy is non-zero but estimated interaction energy is 0) do appear, but the sign of the allosteric modulation is conserved (see Figures 4B,C).

That the model maintains high accuracy for systems with high allosteric efficacy in spite of the two groups of inaccuracy (*i.e*., false positives and true negatives), suggests that this model should reflect many of the qualitative and quantitative properties of actual allosteric systems.

### 2.5. A Relation of AIMs to the Structural Dynamics Analysis of Biomolecular Function

Efforts to identify allosteric sites and channels in the structures of functional biomolecules have utilized estimates of correlation or mutual information between the structural dynamics of known allosteric sites and candidate modulation sites or channels, most often based on the analysis of molecular dynamics (MD) trajectories [33,34,18,19] or elastic network models (ENMs) [35,36]. Equation (43) indicates that structural components that can act as channels will have high effective interaction energy with known allosteric sites (e.g., $u_{C,A}^{\mathrm{int}}$), and the Shannon-Hartley theorem, (8), suggests that the allosteric efficacy can be related to the mutual information via the channel capacity. It is not clear, however, how this relates to the mutual information that is evaluated from an MD simulation. As we and others have used mutual information successfully to interpret the structural dynamics and allostery from MD trajectories [18–20], it is interesting to test the use of mutual information as an identifier of allostery in the context of AIMs. To this end we calculated the *symmetric uncertainty* [37], a normalized variant of the mutual information, between each component in two-component Ising models and two-component non-Ising models, and compared the *symmetric uncertainty* to the absolute interaction energy. The *symmetric uncertainty* (SU) between components is:

(47) $$\text{SU}(X_{i},X_{j})=\frac{2I(X_{i},X_{j})}{H(X_{i})+H(X_{j})}$$

where I is the mutual information:

(48) $$I(X_{i},X_{j})=H(X_{i})+H(X_{j})-H(X_{i},X_{j})$$

and H is the Shannon entropy:

(49) H(X)=−∑p(X)log(p(X))

**Figure 5:**
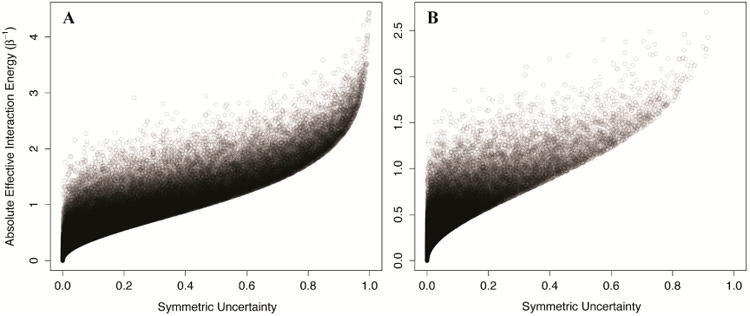
Calculated *mutual information* between the channel and allosteric sites sets a lower bound on the allosteric efficacy. The symmetric uncertainty between the two components is plotted against the absolute effective interaction energy for 100,000 two-component/two-state non-Ising models (**A**), and two-component Ising models (**B**). The systems are generated using energy terms sampled from a normal distribution of mean 0 and standard deviation of 1/β, and the points are plotted with 10% opacity.

We generated 100,000 two-component Ising systems and 100,000 two-component non-Ising systems with energy terms sampled from a normal distribution with mean 0 and standard deviation of 1, and calculated the symmetric uncertainty and allosteric efficacy of each. We find that the symmetric uncertainty enforces a lower limit on the allosteric efficacy, and allosteric efficacy increases with higher symmetric uncertainty (see Figure 5). Thus, mutual information is a good predictor of allosteric activity in the two-state models explored here. The use of mutual information in systems that are not two-state will be discussed further below.

### 2.6. AIMs and Multiple Allosteric Channels

Many proteins have been suggested to have multiple allosteric channels [38]. Assuming that the channels are independent, careful algebra (not shown) reveals that to study the allosteric efficacy of a multi-channel system one can iteratively replace the direct interaction energy term with a direct interaction and indirect interaction of the same effective interaction energy. The effective interaction energy due to multiple independent channels is additive:

(50) $$u_{L,A}^{\mathrm{int}*}=u_{L,A}^{\mathrm{int}}+\sum_{i=1}^{N}u_{L,A}^{\text{indirect},C_{N}}$$

and the allosteric efficacy is then multiplicative:

(51) $$\alpha_{L,A}=\alpha_{L,A|\{C_{1},\ldots ,C_{N}\}}\prod_{i=1}^{N}\alpha_{L,A}^{\text{indirect},C_{N}}$$

This formally obvious result reveals the advantage of multiple channels in an allosteric protein: perturbations such as mutations that disrupt the conformational stability of one channel will not abolish allosteric function completely. Many parallel weak channels introduce significant robustness when compared to the allosterically equivalent single strong channel built in series, because the latter is completely eliminated by disruption of even a single interaction between two of its structural components.

**Figure 6:**
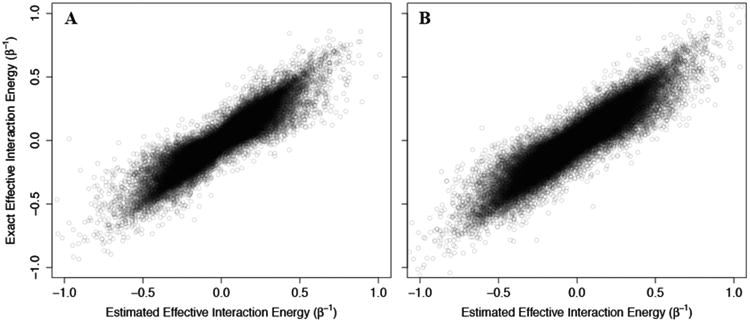
Relation of effective interaction energies in non-Ising two-state systems with multiple independent channels to estimates from the corresponding Ising model. The exact effective interaction energies of 100,000 two-state non-Ising system is plotted against the effective interaction energy estimated using the equations derived for the n-channel Ising model (Equation (51)) for two (**A**), and three (**B**) independent channels. The systems are generated using energy terms sampled from a normal distribution of mean 0 and standard deviation of 1/β, and the points are plotted with 10% opacity.

To test the ability of Equation (51) to reflect accurately the behavior of non-Ising systems, we again constructed 100,000 two- and three-channel non-Ising allosteric systems using the methodology described for single channel systems, and compared the resulting allosteric efficacy to that calculated using (51) (see Figure 6). Again, we find good agreement between the estimates using (51) and the exact calculated efficacies, although the accuracy is slightly reduced as the number of channels increases from two to three.

Because it is unlikely that allosteric proteins consist of absolutely independent channels, we explored the effect of interaction between channels through the use of two AIMs: one two-channel system where both channels provide equal magnitude positive allosteric coupling, and one two-channel system where both channels are of equal magnitude but opposite direction. The allosteric efficacy was calculated for each system as a function of the interaction energy between the two channels of allostery for ligands that are coupled to one, or both channels.

**Figure 7:**
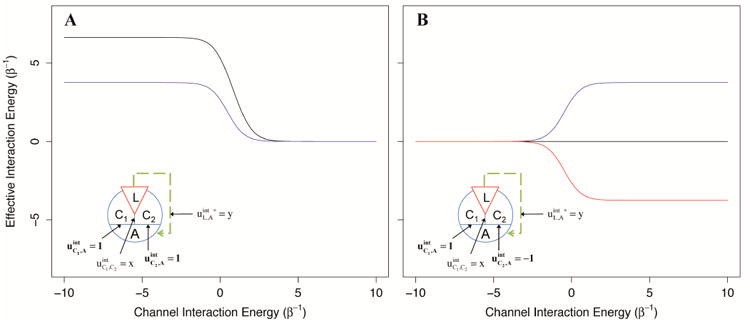
The effective interaction energy of a two-channel AIM as a function of the interaction energy between the channels. (**A**): The two-channel system in which each channel contributes to positive allosteric modulation is shown for a ligand that interacts with one channel (blue) or both channels (black). (**B**): A two-channel system with one positive allosteric channel and one negative allosteric channel is shown for a ligand that interacts only with the positive channel (blue), only with the negative channel (red), or both channels (black). The effect of interactions between channels is seen to modify significantly the allosteric signal transduction.

As depicted in Figure 7, we found that when two channels mediating positive allosteric modulation have a negative interaction energy, the allosteric efficacy of the ligand is increased, even if the ligand only interacts with one channel (Figure 7A). This is not unexpected; the second channel acts as an indirect channel from the first channel to the allosteric site and additionally multiplies the allosteric efficacy of the channel. However, if the ligand interacts with both channels, the allosteric efficacy is not the square of the allosteric efficacy of binding to one channel as would be for two identical, independent channels. This is because the interaction of the ligand with the first channel has already partially shifted the conformational distribution of the second channel, decreasing its channel efficacy by effectively increasing its intrinsic conformational preference (and thus its intrinsic signal-to-noise).

For the second two-channel system, with channels providing allosteric coupling in opposite directions, we find that when the interaction energy between the channels is negative, there is decreased allosteric efficacy for the ligand in either channel, whereas positive interaction energy between the channels leads to increased allosteric efficacy (Figure 7B). From the perspective of the positive channel, if the channels have a negative interaction energy, the second (negative) channel is an indirect channel that flips the sign of the allosteric signal; this leads to reduced overall allosteric efficacy due to negation. However, if the two channels have a positive interaction energy, the signal through the second channel is flipped twice and left unchanged, leading to increased allosteric efficacy. Interestingly, if the ligand interacts with both channels equally, the effective interaction energy from this pair of channels is 0, independent of the interactions between the channels. In a receptor with these characteristics, antagonists could interact with each channel without conformational preference for the channel, or interact with both channels with the same sign, leading to no allosteric signal.

### 2.7. Illustration of AIM-Based Analysis of Allosteric Coupling Mechanisms: The Asymmetric D2 Receptor Homodimeric Signaling Complex

The application of the new formalism based on AIMs was used thus far to represent small, ideal systems in order to extract insights into the physics of allostery on a conceptual level. To examine the practical implementation of AIMs for real allosteric proteins of biological interest, we chose to construct AIMs consisting of a small number of structural components where the numerical calculations of allosteric properties can be performed easily. Such use of AIMs as a coarse-grain level of representation is advantageous in testing hypotheses about the underlying structural mechanisms of real allosteric proteins. This concept is illustrated here with the example of a well-characterized GPCR dimer system.

We constructed a model of asymmetric signaling in the dopamine D2 receptor (D2R) homodimer, based on the structural model of the asymmetric dimer and the constructs used to explore its function that were published recently [39]. Because D2R can signal as both a monomer and a homodimer, a novel experimental construct developed in the Javitch lab [39] was required to make possible the characterization of the dimer as a signaling unit. The results demonstrated experimentally that the D2R homodimer cannot signal through each monomer simultaneously, but instead signals through a single protomer at a time in an asymmetric manner (the signaling protomer will be referred to as “protomer A”). Furthermore, the results indicate that the function of the protomers is characterized by negative cooperativity: the stabilization of the *on* state of the non-signaling monomer (“protomer B”) by agonist biding decreases signaling by protomer A, whereas the stabilization of the *off* state of protomer B by the binding of an inverse agonist increases signaling by protomer A. Lastly, it is shown in [39] that perturbations known to completely disrupt activation in the monomer, including: (i)-ablation of ligand binding, (ii)-removal of intracellular loop 3 (IL3), and (iii)-mutations introduced in (a)-intracellular loop 2 (IL2), (b)-the conserved DRY motif, and (c)-the conserved NPxxY motif, all disrupt activation in the homodimer when applied to protomer A. Unexpectedly, however, the perturbations in (iii) also disrupt activation when applied to protomer B.

**Figure 8:**
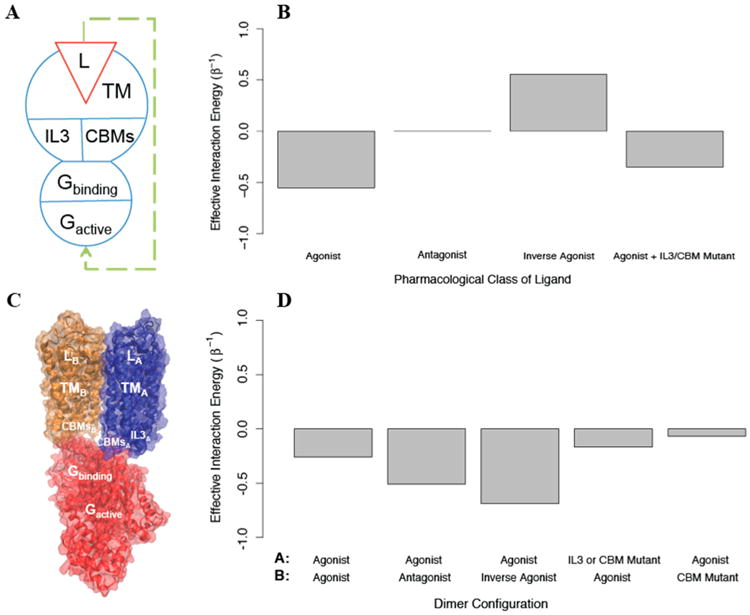
Analysis of the AIM for a well-characterized asymmetric D2 homodimer of the dopamine D2 receptor (D2R). (**A**): The D2R monomer AIM. (**B**): The effective interaction energy calculated for the D2R monomer AIM is presented for ligands that are agonists, antagonists, and inverse agonists, and also for the mutation of either IL3 or the *conserved binding motifs* (CBMs). (**C**): A molecular model of the homodimer obtained as described in the text, is shown with each AIM domain labeled in white on the structural representation. Protomer A is in blue, protomer B is in orange, and the G protein is in red. (**D**): The effective interaction energy for the D2R homodimer AIM is presented for different combinations of the states of protomer A (indicated by **A** in the top row) and those of protomer B in the dimer (**B**, bottom row).

A molecular model of the homodimer complex with the G protein that senses the activation of the receptor was constructed in [39] to explain the experimental results in a structural context. The template for this model was the active state crystal structure of another GPCR, rhodopsin, bound to its G protein, transducin. In this molecular model the interface of the homodimer involves the 4th transmembrane segment (TM4), and the G protein interacts with the signaling protomer A through IL3, IL2, and helix 8 (H8), while protomer B interacts through its IL2 and H8 (see Figure 8). We used AIMs as described below to explore the feasibility of the allosteric properties proposed for this structural model.

Based on the experimental measurements of activation, an AIM representing the homodimer was constructed starting with a model for a signaling monomer (monomer A) and a G protein that can bind this monomer and become activated. Since the IL2, DRY, and NPxxY mutations behave identically in the experiments, we represented all three as a single structural component termed *the conserved binding motifs* (CBMs), due to their role in G protein activation by the GPCR [40–44]. In this AIM (see Figure 8A), the signaling monomer is composed of the following structural components: a ligand that can bind and unbind, a transmembrane domain, and two intracellular regions (IL3 and the CBMs); the G protein is composed of a structural component that can bind and unbind the signaling monomer, and one that can be activated. The conformational energies of the components of each protomer were chosen to prefer the *off* state (u^conf^ =1), and the interaction energies between all components were negative such that they preferred to be in the same state (u^int^ = –1). We find that this coarse grained model responds as expected to agonists, antagonists, and inverse agonists (see Figure 8B). To create a homodimer with negative cooperativity, we then added to the AIM a negative cooperativity between the one monomer that can bind G protein (which is now protomer A) and one that cannot (protomer B), represented as a positive interaction energy between their transmembrane domains (see Figure 8C). We then calculated the allosteric efficacy for the homodimer when protomer A was bound to agonist and protomer B was simultaneously bound to either an agonist, an antagonist, or an inverse agonist. This model reproduces the observed negative cooperativity (See Figure 8D)

To explore the effects of removing IL3 and introducing the CBM mutations, we constructed AIMs with the perturbations modeled as either: i) stabilizing the *off* state of the mutated structural component, ii) stabilizing its *on* state, or iii) reducing the interaction energy between the structural component and the G protein to 0. Modeling the two perturbations in protomer A by imposing (i) or (iii), reduced activation as expected. However, stabilizing the *off* state of IL3 in protomer B increases activation in our model when it should have no effect, indicating that treating the IL3 mutation such that it eliminates interaction between IL3 and the G protein is a better model. On the other hand, treating the CBM perturbation in protomer B as stabilizing the *off* state leads to more activation, so that the effect of the mutation cannot be explained without an interaction between the CBM in protomer B and the G protein. To reconcile these effects in the model, we assumed that protomer B and the G protein bind in a state-independent way (the G protein's state independent binding is represented by $u_{G_{\text{binding}}}^{\text{conf}}$ in the AIM), and modeled the CBM mutation effect as further decreasing state-independent binding. We find that if $u_{G_{\text{binding}}}^{\text{conf}}$ is increased from 1 to 2, allosteric efficacy is reduced (see Figure 8D). The finding that state-independent interactions between the G protein and CBMs on both protomer A and protomer B are required for activation is in full agreement with the structural model of the dimer as presented [39], in which not only protomer A, but also IL2 and H8 from protomer B interact with the G protein directly. As this structural information was not used in the construction of the AIMs, the prediction from the allosteric model underscores the ability of the AIMs-based approach in this illustration to connect the representation of allostery with the structural context of the modeled biomolecular systems.

## 3. Conclusions

We have explored models of biomolecular allostery through the use of Allosteric Ising Models (AIMs) in order to develop a quantitative theoretical description that bridges the features of the structural components and their interactions, to the thermodynamic allosteric parameters. From this perspective, we show first that the allosteric efficacy is the steady state signal-to-noise ratio for the ligand signal through the corresponding noisy receptor. We find that the allosteric efficacy, or the corresponding effective interaction energy, between two allosterically coupled sites can be expressed in terms of the conformational and interaction energies of the constituent parts for many small systems and interaction motifs. This formulation allows us to show that the allosteric efficacy is the product of the indirect allosteric efficacies through independent pathways, suggesting a mechanism by which biomolecular systems have evolved to be robust to mutation. While the equations were derived here using the Ising model to make use of symmetries in the potential energy function, we show that the model can produce good estimates of the allosteric properties of non-Ising two-state pairwise interaction models as well.

A general inference from the use of AIMs as discussed here is that the results can suggest some constraints on the design principles of allosteric proteins. Thus, we find that it is more efficient and more robust to use multiple parallel channels that are individually weak than to use a single series channel that is strong, and that interactions between the parallel channels can additionally increase allosteric efficacy. From a structural perspective it is possible to surmise that α-helices behave as strong serial channels, where as β-sheets behave more like coupled parallel channels that are individually weak. Indeed, it has been shown that significant long-distance correlations exist in β-sheets [45], but little work has been done to study the connection of the properties of these fundamental units of protein structure to their involvement in known allosteric mechanisms. Understanding the allosteric properties of such structural components and common structural motifs from the perspective shown here offers valuable insight into how the wide array of allosteric proteins observed in nature could have been obtained from the limited number of amino acids and folding motifs.

The illustration of the application of AIMs to the D2 receptor homodimer was successful in producing an allosteric model that predicted structural details of molecular interactions. However, it is important to note that the AIM framework assumes that structural components within biomolecular systems exhibit two-state behavior. While this assumption has been used widely in the study of GPCRs and transporters (e.g., the proposed “rocking bundle” mechanism [46,47]), experimental and computational studies indicate [30,48–54] that the character of the conformational space sampled by these molecular machines is not strictly two-state as is often assumed. The principles demonstrated in this manuscript are not mathematically transferable directly to models where structural components require representation by: i)-more than two discrete states, or ii)-continuous states in one or more dimensions. The study of the more complex systems necessitates a more general approach such as the N-body information theoretical analysis we have previously developed [18,55]. We have used such an N-body Information Theory (NbIT) analysis to identify allosteric channels and collective behavior in both transporters [18] and GPCRs [55]. To address the more complex properties of large allosteric systems such as the complex biomolecules responsible for cell function, it may be necessary to formulate a generalization of the NbIT model that allows arbitrary allosteric systems to be constructed and explored in the manner in which the AIMs were analyzed here.

## References

[1] Shi Y (2014). A Glimpse of Structural Biology through X-Ray Crystallography. Cell.

[2] Markwick PRL, Malliavin T, Nilges M (2008). Structural biology by NMR: Structure, dynamics, and interactions. PLoS Comput Biol.

[3] Bai X, McMullan G, Scheres SH (2015). How cryo-EM is revolutionizing structural biology. Trends Biochem Sci.

[4] Ha T (2001). Single-molecule fluorescence resonance energy transfer. Methods.

[5] Sahu ID, McCarrick RM, Lorigan GA (2013). Use of electron paramagnetic resonance to solve biochemical problems. Biochemistry.

[6] Adcock SA, McCammon JA (2006). Molecular dynamics: Survey of methods for simulating the activity of proteins. Chem Rev.

[7] Bahar I, Lezon TR, Yang LW, Eyal E (2010). Global dynamics of proteins: bridging between structure and function. Annu Rev Biophys.

[8] Gether U (2000). Uncovering molecular mechanisms involved in activation of G protein-coupled receptors. Endocr Rev.

[9] Monod J, Changeux JP, Jacob F (1963). Allosteric proteins and cellular control systems. J Mol Biol.

[10] Gunasekaran K, Ma B, Nussinov R (2004). Is allostery an intrinsic property of all dynamic proteins?. Proteins.

[11] Makhlynets OV, Raymond EA, Korendovych IV (2015). Biochemistry.

[12] Hilser VJ, Wrabl JO, Motlagh HN (2012). Structural and energetic basis of allostery. Annu Rev Biophys.

[13] Tsai C, Nussinov R (2014). A Unified View of “How Allostery Works.”. PLoS Comput Biol.

[14] Leff P (1995). The two-state model of receptor activation. Trends Pharmacol Sci.

[15] Fenton AW (2008). Allostery: an illustrated definition for the “second secret of life.”. Trends Biochem Sci.

[16] Shannon CE (1948). A Mathematical Theory of Communication. Bell Syst Tech J.

[17] Shannon CE (1949). Communication in the presence of noise. Proc Inst Radio Eng.

[18] LeVine MV, Weinstein H (2014). NbIT—A New Information Theory-Based Analysis of Allosteric Mechanisms Reveals Residues that Underlie Function in the Leucine Transporter LeuT. PLoS Comput Biol.

[19] Bowman GR, Geissler PL (2012). Equilibrium fluctuations of a single folded protein reveal a multitude of potential cryptic allosteric sites. Proc Natl Acad Sci USA.

[20] Gasper P, Fuglestad B (2012). Allosteric networks in thrombin distinguish procoagulant vs. anticoagulant activities. Proc Natl Acad Sci USA.

[21] Ising E (1925). Beitrag zur theorie des ferromagnetismus. Zeit Phys A Hadron Nucl.

[22] Hopfield J (1982). Neural networks and physical systems with emergent collective computational abilities. Proc Natl Acad Sci USA.

[23] Machta BB, Papanikolaou S, Sethna JP, Veatch SL (2011). Minimal model of plasma membrane heterogeneity requires coupling cortical actin to criticality. Biophys J.

[24] Muñoz V, Thompson PA, Hofrichter J, Eaton WA (1997). Folding dynamics and mechanism of beta-hairpin formation. Nature.

[25] Vorov OK, Livesay DR, Jacobs DJ (2009). Helix/coil nucleation: A local response to global demands. Biophys J.

[26] Vorov OK, Livesay DR, Jacobs DJ (2011). Nonadditivity in conformational entropy upon molecular rigidification reveals a universal mechanism affecting folding cooperativity. Biophys J.

[27] Bray D, Duke T (2004). Conformational spread: the propagation of allosteric states in large multiprotein complexes. Annu Rev Biophys Biomol Struct.

[28] Graham I, Duke T (2005). Dynamic hysteresis in a one-dimensional Ising model: Application to allosteric proteins. Phys Rev E.

[29] Perez DM, Karnik SS (2005). Multiple signaling states of G-protein-coupled receptors. Pharmacol Rev.

[30] Kahsai AW, Xiao K, Rajagopal S, Ahn S, Shukla AK, Sun J, Oas TG, Lefkowitz RJ (2011). Multiple ligand-specific conformations of the β2-adrenergic receptor. Nat Chem Biol.

[31] Urban JD, Clarke WP, Von Zastrow M, Nichols DE, Kobilka B, Weinstein H, Javitch JA, Roth BL, Christopoulos A, Sexton PM, Miller KJ, Spedding M, Mailman RB (2007). Functional Selectivity and Classical Concepts of Quantitative Pharmacology. J Pharmacol Exp Ther.

[32] Kenakin T (2011). Functional selectivity and biased receptor signaling. J Pharmacol Exp Ther.

[33] Sethi A, Eargle J, Black AA, Luthey-Schulten Z (2009). Dynamical networks in tRNA: Protein complexes. Proc Natl Acad Sci USA.

[34] Pandini A, Fornili A, Fraternali F, Kleinjung J (2012). Detection of allosteric signal transmission by information-theoretic analysis of protein dynamics. FASEB J.

[35] Ming D, Wall ME (2005). Allostery in a coarse-grained model of protein dynamics. Phys Rev Lett.

[36] Su JG, Qi LS, Li CH, Zhu YY, Du HJ, Hou YX, Hao R, Wang JH (2014). Prediction of allosteric sites on protein surfaces with an elastic-network-model-based thermodynamic method. Phys Rev E.

[37] Witten IH, Eibe F, Hall MA (2005). Data Mining Practical Machine Learning Tools and Techniques.

[38] Del Sol A, Tsai CJ, Ma B, Nussinov R (2009). The origin of allosteric functional modulation: multiple pre-existing pathways. Structure.

[39] Han Y, Moreira IS, Urizar E, Weinstein H, Javitch JA (2009). Allosteric communication between protomers of dopamine class A GPCR dimers modulates activation. Nat Chem Biol.

[40] Moro O, Lameh J, Högger P, Sadée W (1993). Hydrophobic amino acid in the i2 loop plays a key role in receptor-G protein coupling. J Biol Chem.

[41] Ballesteros JA, Jensen AD, Liapakis G, Rasmussen SGF, Shi L, Gether U, Javitch JA (2001). Activation of the β2-Adrenergic Receptor Involves Disruption of an Ionic Lock between the Cytoplasmic Ends of Transmembrane Segments 3 and 6. J Biol Chem.

[42] Fritze O, Filipek S, Kuksa V, Palczewski K, Hofmann KP, Ernst OP (2003). Role of the conserved NPxxY(x)5,6F motif in the rhodopsin ground state and during activation. Proc Natl Acad Sci USA.

[43] Shan J, Khelashvili G, Mondal S, Mehler EL, Weinstein H (2012). Ligand-Dependent Conformations and Dynamics of the Serotonin 5-HT 2A Receptor Determine Its Activation and Membrane-Driven Oligomerization Properties. PLoS Comput Biol.

[44] Han DS, Wang SX, Weinstein H (2008). Active state-like conformational elements in the beta2-AR and a photoactivated intermediate of rhodopsin identified by dynamic properties of GPCRs. Biochemistry.

[45] Fenwick RB, Orellana L, Esteban-Martín S, Orozco M, Salvatella X (2014). Correlated motions are a fundamental property of β-sheets. Nat Commun.

[46] Forrest LR, Rudnick G (2009). The rocking bundle: a mechanism for ion-coupled solute flux by symmetrical transporters. Physiology (Bethesda).

[47] Forrest LR, Zhang YW, Jacobs MT, Gesmonde J, Xie L, Honig BH, Rudnick G (2008). Mechanism for alternating access in neurotransmitter transporters. Proc Natl Acad Sci USA.

[48] Nygaard R, Zou Y, Dror RO, Mildorf TJ, Arlow DH, Manglik A, Pan AC, Liu CW, Fung JJ, Bokoch MP, Thian FS, Kobilka TS, Shaw DE, Mueller L, Prosser RS, Kobilka BK (2013). The dynamic process of β(2)-adrenergic receptor activation. Cell.

[49] Provasi D, Artacho MC, Negri A, Mobarec JC, Filizola M (2011). Ligand-induced modulation of the free-energy landscape of G protein-coupled receptors explored by adaptive biasing techniques. PLoS Comput Biol.

[50] Kazmier K, Sharma S, Quick M, Islam SM, Roux B, Weinstein H, Javitch JA, McHaourab HS (2014). Conformational dynamics of ligand-dependent alternating access in LeuT. Nat Struct Mol Biol.

[51] Shi L, Quick M, Zhao Y, Weinstein H, Javitch JA (2008). The mechanism of a neurotransmitter:sodium symporter--inward release of Na^+^ and substrate is triggered by substrate in a second binding site. Mol Cell.

[52] Zhao C, Stolzenberg S, Gracia L, Weinstein H, Noskov S, Shi L (2012). Ion-controlled conformational dynamics in the outward-open transition from an occluded state of LeuT. Biophys J.

[53] Zhao Y, Terry D, Shi L, Weinstein H, Blanchard SC, Javitch JA (2010). Single-molecule dynamics of gating in a neurotransmitter transporter homologue. Nature.

[54] Zhao Y, Terry DS, Shi L, Quick M, Weinstein H, Blanchard SC, Javitch JA (2011). Substrate-modulated gating dynamics in a Na+-coupled neurotransmitter transporter homologue. Nature.

[55] LeVine M, Perez-Aguilar J, Weinstein H N-body Information Theory (NbIT) Analysis of Rigid-Body Dynamics in Intracellular Loop 2 of the 5-HT2A Receptor.

